# Pelvic Surgical Site Infections in Gynecologic Surgery

**DOI:** 10.1155/2015/614950

**Published:** 2015-02-18

**Authors:** Mark P. Lachiewicz, Laura J. Moulton, Oluwatosin Jaiyeoba

**Affiliations:** ^1^Department of Gynecology and Obstetrics, Emory University School of Medicine, 1639 Pierce Drive, 4th Floor WMB, Atlanta, GA 30322, USA; ^2^Cleveland Clinic Foundation, Women's Health Institute, Cleveland, OH, USA

## Abstract

The development of surgical site infection (SSI) remains the most common complication of gynecologic surgical procedures and results in significant patient morbidity. Gynecologic procedures pose a unique challenge in that potential pathogenic microorganisms from the skin or vagina and endocervix may migrate to operative sites and can result in vaginal cuff cellulitis, pelvic cellulitis, and pelvic abscesses. Multiple host and surgical risk factors have been identified as risks that increase infectious sequelae after pelvic surgery. This paper will review these risk factors as many are modifiable and care should be taken to address such factors in order to decrease the chance of infection. We will also review the definitions, microbiology, pathogenesis, diagnosis, and management of pelvic SSIs after gynecologic surgery.

## 1. Introduction

The development of surgical site infection (SSI) results in significant patient morbidity. Postoperative infection remains the most common complication of surgical procedures in gynecology [1]. Prior to the advent of routine antimicrobial prophylaxis, pelvic infection rates after vaginal hysterectomy were as high as 33%, with pelvic cellulitis seen most frequently [2]. The widespread implementation of antibiotic prophylaxis prior to surgery, as well as recognition of modifiable risk factors for postoperative infection, has led to a significant reduction in postoperative infection rates. In a recent cross-sectional analysis of the 2005–2009 American College of Surgeon's National Surgical Quality Improvement Program participant data files, there was a 2.7% occurrence for superficial, deep, and organ space infections after hysterectomy [3]. This paper will review the definitions, microbiology, pathogenesis, and risk factors of pelvic SSIs after gynecologic surgery with a focus on vaginal cuff cellulitis, pelvic cellulitis, and pelvic abscesses. We will also review the diagnosis and management of these conditions.

## 2. Definition of Pelvic Infections after Gynecologic Surgery

The Centers for Disease Control and Prevention (CDC) defines a SSI as an infection occurring within 30 days of an operation occurring in one of 3 locations: superficial at the incision site, deep at the incision site, or in other organs or spaces opened or manipulated during an operation [4].

### 2.1. Superficial Incisional SSI Includes Vaginal Cuff Cellulitis

Infection involves only the skin and subcutaneous tissue of the incision and at least one of the following:

1. purulent drainage with or without laboratory confirmation, from the superficial incision;
2. organisms isolated from an aseptically obtained culture of fluid or tissue from the superficial incision;
3. at least one of the following signs or symptoms of infection: pain or tenderness, localized swelling, redness, or heat and superficial incision being deliberately opened by surgeon, unless incision is culture-negative.

### 2.2. Deep Incisional SSI Includes Pelvic Cellulitis

The infection involves deep soft tissue (e.g., fascia, muscle) of the incision and at least one of the following:

1. purulent drainage from the deep incision but not from the organ/space component of the surgical site;
2. a deep incision which spontaneously dehisces or is deliberately opened by a surgeon when the patient has at least one of the following signs or symptoms: fever (>38°C), localized pain, or tenderness, unless incision is culture-negative;
3. an abscess or other evidence of infection involving the deep incision being found on direct examination, during reoperation, or by histopathologic or radiologic examination.

### 2.3. Organ/Space SSIs Include Adnexal Infections and Pelvic Abscesses

Infection involves any part of the anatomy (e.g., organs and spaces) other than the incision that was opened or manipulated during an operation and at least one of the following:

1. purulent drainage from a drain that is placed through a stab wound into the organ/space;
2. organisms isolated from an aseptically obtained culture of fluid or tissue in the organ/space;
3. an abscess or other evidence of infection involving the organ/space that is found on direct examination, during reoperation, or by histopathologic or radiologic examination [4].

## 3. Microbiology and Pathogenesis

Microbial contamination of the surgical site by endogenous skin or vaginal flora is a fundamental precursor to postoperative SSI. The risk of infection is significantly elevated when there is an increased concentration and virulence of contaminating bacteria. Quantitatively, it has been shown that the risk for developing an infection increases markedly if the operating site is contaminated with >10^5^ microorganisms per gram of tissue. However, in the presence of foreign bodies, such as suture material, this required inoculum decreases to 10^3^ microorganisms per gram of tissue [5–9]. Conversely, both systemic and local host immune defense mechanisms function to contain inoculated bacteria and prevent infection. Prophylactic antibiotics in the tissue augment the natural host immunity.

For most SSIs, the source of pathogens is the endogenous flora of the patient's skin, which consists of predominantly aerobic gram-positive cocci [7, 10]. However, gynecologic procedures pose a unique challenge in that potential pathogenic microorganisms may come from the skin or ascend from the vagina and endocervix to the operative sites, including the abdominal incision and vaginal cuff. The endogenous vaginal flora is a complex and dynamic mix of pathogenic and nonpathogenic bacteria composed of facultative and obligate anaerobic gram-positive and gram-negative species. Therefore, gynecologic SSIs are more likely to be polymicrobial and may include gram-negative bacilli, enterococci, group B streptococci, and anaerobes as a result of incisions involving the vagina and perineum. If the balance of pathogenic to nonpathogenic bacteria is disrupted, these bacteria can gain access to the sterile tissue of the pelvis and can lead to infection. Bacterial vaginosis (BV) is a well-documented risk factor for SSI after pelvic surgery, specifically vaginal cuff cellulitis. BV is a complex alteration in the vaginal flora resulting in an increased concentration of potentially pathogenic anaerobic bacteria at levels reported at 1000–10000-fold greater than normal [8, 10, 11].

The development of infection results from ineffective host defense mechanisms and insufficient antibiotic prophylaxis in the setting of a high bacterial inoculum in virulent species [9]. Microorganisms produce toxins and other virulence factors that increase their ability to invade, cause damage to, and survive within or on host tissue. In the case of postoperative pelvic abscess, it is hypothesized that blood, lymphatic and serous fluid, necrotic debris, and fibrillar hemostats can accumulate in the lower pelvis and around the vaginal vault and produce a simple fluid collection. This fluid collection can subsequently become infected through contamination from the skin, through the vaginal opening, or after bowel resections and may result in formation of pelvic abscess. The incidence of pelvic abscess in gynecologic surgery is estimated at 1% [8, 11–14].

## 4. Risk Factors

Multiple host and surgical factors have been identified that increase the risk of infectious sequelae after pelvic surgery. Many of these risk factors are modifiable and care should be taken to address such factors in order to decrease the chance of infection.

## 5. Host Risk Factors

The preoperative evaluation of a patient provides an excellent opportunity to evaluate for the presence of modifiable and nonmodifiable host risk factors for SSIs. A cross-sectional analysis of the 2005–2009 American College of Surgeon's National Surgical Quality Improvement Program patient files identified many risk factors for SSI [3]. Obesity significantly influences risk for gynecologic and obstetrical SSI, specifically in patients with a BMI of greater than 30 or with depth of subcutaneous tissue greater than 2 cm. Diabetes mellitus is associated with elevated risk of infection postoperatively, particularly in patients with perioperative serum glucose levels greater than 150 mg/dL and preoperative hemoglobin HbA_1c_ greater than 6.5%. Patients with preexisting medical illness such as diabetes should be medically optimized prior to surgery. Preoperative anemia and history of cerebrovascular accidents were also associated with deep and organ space SSI [3]. There are several other well-documented risk factors for SSI within the surgical literature including tobacco use, corticosteroid use, malnutrition, and increased age [15–19]. History of radiation to the surgical site also elevates risk of infection [7]. Bacterial vaginosis is associated with a significantly elevated risk of postoperative infections, specifically vaginal cuff cellulitis. Therefore preoperative screening and treatment is an important deterrent to postoperative infection [11, 20]. Colonization or infection with other organisms at the time of operation including Group B streptococci,* Trichomonas*,* S. aureus* nasal carriage, and history of MRSA have been demonstrated to elevate risk [21–23]. Prolonged preoperative hospitalization should be avoided to decrease the risk of patients becoming colonized with nosocomial bacteria, as these microorganisms tend to be more resistant to antibiotics compared to endogenous bacteria [24].

## 6. Surgical Risk Factors

### 6.1. Preoperative Risk Factors

Prophylactic antibiotics decrease the bacterial inoculum burden on the skin and make the operative site less hospitable to the growth of bacteria. Furthermore, antibiotics concentrate in white blood cells resulting in enhanced phagocytosis of pathogenic bacteria [2]. The antibiotic of choice for prophylaxis should have broad coverage, be inexpensive, and be easy to administer. Cefazolin meets this criterion. Antibiotics should be administered within an hour of incision. Current recommendations for pre-op antibiotics were recently revised. Patients undergoing an extended procedure (≥3 hours) or with a total blood loss ≥1500 mL should receive a second dose of antibiotic. Obese women with a weight of ≥120 kg should receive an increased dose of antibiotics. For example, cefazolin should be increased to 3 grams in these patients, as opposed to the standard 2 grams in women with a weight of less than 120 kg. Recommended antimicrobial prophylaxis regimens, doses, and redosing intervals for gynecologic surgery are listed in Tables 1 and 2 [25].

Preoperative preparation of the skin and vagina with Povidone-Iodine or chlorhexidine gluconate is universally recommended to reduce risk of postoperative cuff cellulitis and abscess. Despite concerns about using chlorhexidine gluconate to prep the vagina, the American Congress of Obstetricians and Gynecologists (ACOG) recently supported the use of chlorhexidine gluconate to prep the vagina [26]. A recent analysis demonstrated an elevation in superficial SSI with route of hysterectomy, with the abdominal method associated with higher rate of infection compared to the vaginal approach. Comparatively, the rates of deep superficial and organ space infections were similar regardless of surgical approach [3].

### 6.2. Intraoperative Risk Factors

Intraoperative events including increased blood loss greater than 500 mL, prolonged surgical procedure greater than 140 minutes, and blood transfusion are associated with development of deep and organ space SSI [3, 13, 23]. Staple closure was associated with significantly increased wound infectious morbidity compared to closure with sutures in a randomized control trial by Figueroa et al. [27]. Fibrillar oxidized regenerated cellulose may contribute to pelvic abscess formation. The hemostatic agent can trap tissue debris, protect bacteria from host-defense mechanisms, and with unopposed bacterial proliferation lead to abscess formation [8].

For operations performed laparoscopically, direct trocar insertion and open technique may confer a lower postinfection rate than entry with the Veress needle [28]. Single-port laparoscopic hysterectomy appears to have a lower infection rate than traditional four-port laparoscopic hysterectomy [29]. Removal of fallopian tubes at the time of hysterectomy may also significantly decrease the risk of infectious complications [30]. Robotic-assisted procedures do not appear to confer any advantage versus convention laparoscopy from an infectious standpoint [31].

Patients undergoing pelvic lymphadenectomy, para-aortic lymphadenectomy, splenectomy, bowel resection, or pelvic exenteration for surgical treatment of gynecologic malignancies are associated with increased risk of deep superficial and organ space SSIs [8, 23].

### 6.3. Postoperative Risk Factors

Postoperative anemia has been defined as a significant risk factor for all classifications of SSI in obstetrical and gynecologic surgery [32]. Poor glucose control, defined as levels greater than 200 mg/dL within the first 48 hours postoperatively, increased the likelihood of pelvic infections [33]. Increased length of duration of hospital stay perioperatively has also been correlated with increased incidence of SSIs [7].

## 7. Clinical Features and Management of SSI

Typically, postoperative pelvic infections, including vaginal cuff cellulitis and pelvic abscess, present with complaint of pelvic pain with fever with associated tachycardia and leukocytosis. The approach to management depends on the clinical status of the patient and characteristics of the pelvic infection. Appropriate antimicrobial therapy of pelvic abscesses includes coverage against aerobic and anaerobic bacteria with an ability to penetrate abscess cavities while remaining stable in an acidic, hypoxic environment, typical of an abscess [34].

## 8. Vaginal Cuff Cellulitis

Vaginal cuff cellulitis is an infection of the superficial tissues at the vaginal surgical margin after vaginal hysterectomy. Patients typically present after hospital discharge with moderate, but increasing, lower abdominal pain with purulent yellow vaginal discharge. Physical examination will reveal the vaginal surgical margin to be tenderness out of proportion to what is expected with hyperemia and edema. The adnexa and parametria are nontender. Treatment is outpatient oral antibiotic therapy with a single broad-spectrum agent with close follow-up to assure treatment efficacy [7, 9, 35].

Recommended regimens for treatment of vaginal cuff cellulitis include

- amoxicillin/clavulanate 875/125 mg PO bid,
- ciprofloxacin 500 mg po bid with metronidazole 500 mg po bid,
- TMP-SMX DS po bid with metronidazole 500 mg po bid [7].

## 9. Pelvic Cellulitis

Patient with pelvic cellulitis typically presents 5 to 10 days after surgery with fever, vague abdominal pain, or the sensation of pelvic fullness. Associated symptoms may include anorexia, but they typically do not have gastrointestinal or urinary complaints. Physical examination will reveal regional tenderness to palpation, with edema in the absence of masses or peritoneal signs. Ultrasound will demonstrate no masses. Hospitalization is indicated and patients should be treated with an intravenous broad-spectrum antibiotic regimen until they have been afebrile for 24–48 hours and may be discharged on an oral antibiotic regimen with coverage for gram-positive, gram-negative, and anaerobic bacteria [7, 36]. Antibiotics regimens are the same as for pelvic abscesses and are discussed below.

## 10. Pelvic Abscess

Pelvic abscesses are a rare but serious complication of pelvic surgery occurring when pelvic cellulitis or pelvic hematoma spread into the parametrial soft tissue [9]. Pelvic abscess symptoms mirror that of pelvic cellulitis, with the addition of a palpable mass corresponding to the collection of infected fluid or visualization of the fluid collection by ultrasonography, computed tomography (CT), or magnetic resonance image (MRI). As soon as the diagnosis is reached, broad-spectrum antibiotics should be administered intravenously until patient is afebrile for 48–72 hours. Drainage via ultrasound or CT guidance, laparoscopy, or laparotomy may be required [7, 36].

## 11. Antibiotic Therapy

Candidates for antibiotic therapy alone may be recommended for the following women:

- hemodynamically stable,
- no signs of sepsis or rupture of the abscess,
- adequate response to antibiotic therapy,
- pelvic abscess ≤8 cm in diameter [37].

Patients are empirically started on antibiotics and one intravenous antibiotic regimen that has been studied extensively is the combination of clindamycin (900 mg every 8 hours) or metronidazole (500 mg every 12 hours) plus penicillin (5 million units every 6 hours) or ampicillin (2 g every 6 hours) plus gentamicin (5 mg/kg ideal body weight every 24 hours). Aztreonam (2 g every 8 hours) is substituted for gentamicin in patients who have renal impairment [38, 39].

Additional agents with therapeutic utility include single agent treatment with an extended-spectrum cephalosporin (e.g., cefoxitin, cefotetan, cefotaxime, and ceftizoxime), an extended-spectrum penicillin (e.g., piperacillin-tazobactam), beta-lactamase inhibitors plus a beta-lactam (e.g., ticarcillin-clavulanate), and carbapenems (ertapenem or meropenem) [38, 39].

While treatment regimens containing aminoglycosides have been used effectively in the treatment of pelvic abscesses, this class of antibiotics has their activity reduced at low pH, at low oxygen tension, and in the presence of drug-binding purulent debris [40]. Ceftriaxone is a third-generation cephalosporin that has a much higher serum level to minimum inhibitory concentration ratio when compared with aminoglycosides. It has high antibacterial potency, broad spectrum of activity, low potential for toxicity, and favorable pharmacokinetics. Furthermore, ceftriaxone is highly protein bound causing it to have the longest half-life of amongst the drugs in its class which translates to convenient once daily dosing [41].

Clindamycin is actively transported into polymorphonuclear leukocytes and macrophages and has been demonstrated in relatively high concentrations, compared with peak serum levels, in experimental abscesses [42]. An important use of clindamycin is in treatment of infections likely to involve* B. fragilis* or other penicillin-resistant anaerobic bacteria. It is beneficial where there is spillage of fecal flora, associated with tissue damage. Studies suggest that clindamycin decreases the likelihood of abscess formation involving fecal organisms like* B. fragilis* but must be coadministered with an aminoglycoside, a third-generation cephalosporin like ceftriaxone or aztreonam, because additional activity is required against* Enterobacteriaceae*. Given reports of increasing resistance of Bacteroides species to clindamycin, use of clindamycin may be entirely replaced in the future with other agents, such as metronidazole, where resistant strains are rare [34, 43].

*Based on the available literature, we recommend metronidazole (500 mg every 12 hours) plus ceftriaxone (2 g every 24 hours) as our first line treatment for pelvic abscesses.*

Parenteral antibiotics should be continued until the patient is afebrile for 24–48 hours. Patient should subsequently receive oral antibiotics to complete a 14-day course of therapy. Patients should be switched to antibiotics based on culture and sensitivities when available. Combination of oral metronidazole (500 mg every 12 hours) and trimethoprim/sulfamethoxazole (160/800 mg every 12 hours) or amoxicillin/clavulanate (875/125 mg every 12 hours) monotherapy can be used due to excellent polymicrobial coverage [39].

## 12. Drainage or Surgical Therapy

Traditionally, the treatment algorithm was to start antibiotics and monitor a patient for improvement. Recent evidence suggests it is acceptable or may be of benefit to choose primary drainage with concomitant antibiotic therapy or after initiation of antibiotic therapy. Routine drainage of pelvic abscesses can decrease prolonged hospitalizations and improve reproductive outcomes [37, 44]. This strategy has been recommended as a first-line procedure especially in women of reproductive age. Drainage at an early time-point after admission to hospital is also more efficient than medical treatment alone with regard to treatment success in addition to decreasing mean hospital stay [45, 46].

Regardless, drainage should be performed if an adequate response to antibiotic therapy is not registered within 2-3 days or if the pelvic abscess is >8 cm [37, 47].

Criteria for failure may include the following:

- Patients with no radiological reduction in abscess size. Greater than a 50% reduction should be seen.
- Patients whose abscess progressively increased in size.
- New onset fever or persistent fevers.
- Clinical deterioration with persistent or worsening abdominal/pelvic tenderness despite appropriate antibiotic therapy.
- Patients meeting criteria for sepsis. Septic patients should be continued on antibiotics and taken to the operating room for emergent operative treatment.
- Ruptured or suspected intra-abdominal rupture of abscess. Abscess rupture is life-threatening emergency that can result in sepsis and septic shock. Ruptured abscess should be treated immediately. Surgical intervention is advocated in these patients to improve their outcome. These patients should also be continued on antibiotics and taken to the operating room for emergent operative treatment.

Drainage can be performed by laparoscopy and has several advantages compared to laparotomy [44]. However, CT- or ultrasound-guided drainage in combination with antibiotics has emerged as a preferred alternative approach in hemodynamically stable patients with excellent results, even with large abscesses [48, 49]. This approach has several advantages compared to antibiotic therapy alone as well as laparoscopy and antibiotics, including no required anesthesia, immediate pain relief, and reduced duration of hospital stay [46, 48]. Therefore, our preferred method of drainage is percutaneous drainage guided by CT or ultrasound. Pelvic cuff abscesses can also be drained by ultrasound guided transvaginal aspiration with excellent outcomes [50, 51]. If the abscess is accessible via the vaginal apex or cul-de-sac, a transvaginal aspiration may be a more appropriate option. While colpotomy or placement of a transvaginal drain (i.e., Foley or Malecot) has been used successfully in the past, the complication rate may be higher than aspiration alone without additional benefit [52–55]. Exudate should be sent for gram stain, culture, and sensitivity. Antibiotics should be adjusted based on culture and sensitivities.

*Based on the above studies, our recommendations regarding drainage are the following: pelvic abscess >8 cm should be drained in addition to the administration of empiric parenteral antibiotics; cultures and sensitivities should be obtained; early drainage of a pelvic abscess is safe, improves outcomes, reduces hospitalization, and is appropriate for the clinician to consider as primary therapy; and the preferred method for drainage is percutaneous by the interventional radiologist or transvaginally if the patient is hemodynamically stable.*

## 13. Summary and Recommendations

- Postoperative cuff and pelvic abscesses are among the most common complications of gynecologic surgeries.
- Evaluation for preoperative and postoperative risk factors and managing modifiable risk factors can decrease infection rates.
- Pelvic abscesses are usually polymicrobial and contain both aerobic and anaerobic bacteria.
- Pelvic cellulitis typically presents 5 to 10 days after surgery with fever, vague abdominal pain, or the sensation of pelvic fullness. Pelvic abscess symptoms mirror that of pelvic cellulitis with the addition of a palpable mass corresponding to the collection of infected fluid or radiographic evidence of abscess.
- Approach to management depends on the clinical status of the patient and characteristics of the pelvic abscess. Treatment with antibiotics alone is appropriate for women who meet the following criteria: being hemodynamically stable, having pelvic abscess <8 cm in diameter, and having adequate response to antibiotic therapy.
- Our recommended antibiotic regimen for pelvic abscesses is metronidazole (500 mg every 12 hours) plus ceftriaxone (2 g every 24 hours) (Table 3).
- Minimally invasive drainage, laparoscopy, or exploratory laparotomy may be required in women with abscesses >8 cm or who show no signs of improvement but are not worsening clinically.
- Clinically worsening patients, suspected rupture, and septic patients require immediate laparotomy which may be life-saving.
- Duration of therapy is for at least 14 days or more depending on resolution of the pelvic abscess.

## Figures and Tables

**Table 1:** Antimicrobial prophylaxis in gynecologic surgery.

Type of procedure	Recommended agents	Alternative agents in pts with *β*-lactam allergy
Hysterectomy	Cefazolin, cefotetan, cefoxitin, or ampicillin-sulbactam^[a]^	Clindamycin or vancomycin + aminoglycoside^[b]^; or aztreonam alone; or fluoroquinolone alone^[a,c]^; or metronidazole + aminoglycoside or fluoroquinolone

Laparoscopic procedure, low-risk	None	None

Laparoscopic procedure, high-risk^[d]^	Cefazolin, cefoxitin, cefotetan, ampicillin-sulbactam^[a]^	Clindamycin or vancomycin + aminoglycoside^[b]^; or aztreonam alone; or fluoroquinolone alone^[a,c]^; or metronidazole + aminoglycoside or fluoroquinolone

Clean-contaminated cancer surgery	Cefazolin + metronidazole, cefuroxime + metronidazole, ampicillin-sulbactam^[a]^	Clindamycin

Adapted from  [25].

^[a]^Due to increasing resistance of *Escherichia coli* to fluoroquinolones and ampicillin-sulbactam, local population susceptibility profiles should be reviewed prior to use.

^[b]^Gentamicin.

^[c]^Ciprofloxacin or levofloxacin; fluoroquinolones are associated with an increased risk of tendonitis and tendon rupture in all ages. However, this risk would be expected to be quite small with single-dose antibiotic prophylaxis. Although the use of fluoroquinolones may be necessary for surgical antibiotic prophylaxis in some children, they are not drugs of first choice in the pediatric population due to an increased incidence of adverse events as compared with controls in some clinical trials.

^[d]^Factors that indicate a high risk of infectious complications include emergency procedures, diabetes, long procedure duration, age of >70 years, American Society of Anesthesiologists classification of 3 or greater, pregnancy, immunosuppression, and insertion of prosthetic device.

**Table 2:** Recommended doses and redosing intervals for commonly used antimicrobials for surgical prophylaxis for gynecological procedures^[a]^.

Antimicrobial	Recommended dose	Half-life (hours)	Recommended redosing interval (hours)^[b]^
Ampicillin-sulbactam	3 g (ampicillin 2 g/sulbactam 1 g)	0.8–1.3	2
Aztreonam	2 g	1.3–2.4	4
Cefazolin	2 g, 3 g for pts weighing ≥120 kg	1.2–2.2	4
Cefuroxime	1.5 g	1-2	4
Cefoxitin	2 g	0.7–1.1	2
Cefotetan	2 g	2.8–4.6	6
Ciprofloxacin	400 mg	3–7	NA
Clindamycin	900 mg	2–4	6
Gentamicin	5 mg/kg based on dosing weight (single dose)^[c]^	2-3	NA
Levofloxacin	500 mg	6–8	NA
Metronidazole	500 mg	6–8	NA

Adapted from [25].

^[a]^Dosing and redosing interval for adult patients with normal renal function.

^[b]^Redosing in the operating room is recommended at an interval of approximately two times the half-life of the agent in patients with normal renal function. Recommended redosing intervals marked as “not applicable” (NA) are based on typical case length; for unusually long procedures, redosing may be needed.

^[c]^ln general, gentamicin for surgical antibiotic prophylaxis should be limited to a single dose given preoperatively. Dosing is based on the patient's actual body weight. If the patient's actual weight is more than 20% above ideal body weight (IBW), the dosing weight (DW) can be determined as follows: DW = IBW + 0.4(actual weight − IBW).

**Table 3:** Recommended antibiotic regimen for pelvic infections after gynecologic surgery.

Infection type	Antimicrobials	Duration of treatment
Vaginal cuff cellulitis	Oral regimen Amoxicillin/clavulanate 875/125 mg q 12 h OR Ciprofloxacin 500 mg q 12 h PLUS Metronidazole 500 mg q 12 h OR Trimethoprim/sulfamethoxazole 160/800 mg q 12 h PLUS Metronidazole 500 mg q 12 h	7–14 days

Pelvic cellulitis and pelvic abscesses^[a]^	Parenteral regimens Clindamycin 900 mg q 8 h or Metronidazole 500 mg q 12 h PLUS Ceftriaxone 2 g q 24 h OR Clindamycin 900 mg q 8 h or Metronidazole 500 mg q 12 h PLUS Penicillin 5 million u q 6 h or Ampicillin 2 g q 6 h PLUS Gentamicin 5 mg/kg IBW q 24 h OR Aztreonam 2 g q 8 h^[b]^ Oral regimen Metronidazole 500 mg q 12 h PLUS Trimethoprim/sulfamethoxazole 160/800 mg q 12 h OR Amoxicillin/clavulanate (875/125 mg q 12)	14 days

^[a]^Parenteral antibiotics should be continued until the patient is afebrile for 24–48 hours. Patient should subsequently receive oral antibiotics to complete 14-day course of antibiotics.

^[b]^Aztreonam 2 g q 8 h may be substituted for gentamicin in patients who have renal impairment.
